# Supplementary material for: Molecular dynamics simulation of proton-transfer coupled rotations in ATP synthase FO motor
Source: Sci Rep. 2020 May 19;10:8225. doi: 10.1038/s41598-020-65004-1 (PMC7237500; doi:10.1038/s41598-020-65004-1)
Supplement: Supplementary file 1 — Supplementary information. [file 41598_2020_65004_MOESM1_ESM.docx]

Title

Molecular dynamics simulation of proton-transfer coupled rotations in ATP synthase F_O_ motor

**Authors**

Shintaroh Kubo^1†^, Toru Niina^1†^, Shoji Takada^1*^

**Affiliations**

^1.^ Department of Biophysics, Graduate School of Science, Kyoto University, Kyoto 606-8502, Japan

^†.^ These authors contributed equally to this work.

^*.^ The corresponding author: takada@biophys.kyoto-u.ac.jp

Supplementary Materials

**Section S1. Analyzing Free Energy Differences Between Different Protonation States.**

We compared the free energy curves in different protonation states. We chose two representative pathways of proton-transfer coupled rotary motions observed in our MC/MD simulation described above and picked 8 protonation states from the pathways. Then we carried out 10 independent 10^8^ MD simulations for each state with 8 fixed protonation states. Since we need to fix the states of each protonatable site while the MD simulation, the states of aE223 and aE162 are fixed in this simulation. We chose the protonation states of the a-subunit to make the total number of protons kept upon the transitions along each pathway.

Using those samples, we employed the Bennett acceptance ratio method [50] and calculated free energy differences between the states that are next to each other in the pathways. For this calculation, we applied the protonation-state dependent energy function. Applying the method to each pair of states, we evaluated the free energy differences of each transition in the pathways. Because the effect of proton exchanges between the a-subunit and bulk are not considered in the procedure, the net free energy differences after the step of 36 degrees of rotation became zero in each proton transfer pathways, confirming the accuracy of our calculations. To consider the stabilization of transmembrane proton transfer, we reinterpreted the proton movement in the step in which a proton is exchanged between aE223 and aE162 as proton exchange between each residue and the bulk. As a result, we obtained the free energy differences between the first state and the state after 36 degrees of rotation as -4.9 kcal/mol that is the same value as the stabilization by one proton transfer from IMS to matrix in our standard setup.

Supplementary Figures

**Fig. S1. Details in the simulation setup. (A)** The schematic view of our implicit membrane potential, $V_{mem}(\mathbf{R},\mathbf{H}^{+})$. We applied a free energy cost to the charged, i.e., deprotonated residues. If the residues are protonated, the free energy cost vanishes. **(B)** The illustration of the geometric parameters used in the kinetic weigh $w$. The first vector $v_{1}$ connects the middle point of the adjacent residues on the c_10_-ring (left gray circles) and the protonation site on c_10_-ring (green). The second vector $v_{2}$ connects the protonation site on c_10_-ring (green) and the site on a-subunit (red).

**Fig. S2. The trajectories of the WT F_O_ with non-zero proton-motive-force in the ATP hydrolysis mode. (A)** Time courses of the cumulative rotation angle (top) and the number of protons that across all the way from IMS side to matrix side (bottom) for 10 trajectories. The negative rotation angle means the clockwise rotation corresponding to proton pumping. Note that the negative number of protons means the protons transfer in the opposite way, from matrix side to IMS side. **(B)** Probability distribution of the rotation angle in all the trajectories is plotted in a negative logarithmic scale.

**Fig. S3. Comparison of the mechanisms between the aR176A mutant and the WT F_O_'s in the ATP synthesis mode. (A)** The same as the one shown in Fig.2D. **(B)** The result of the same analysis for the aR176A mutant. The magenta line indicates Path7.

**Fig. S4. The number of revolutions per trajectory under different external torque.** The number of revolutions in one trajectory is plotted. Between $43.0 pN\cdot nm$ and $51.6 pN\cdot nm$, the number of rotations turned from positive to negative.

**Fig. S5. The effect of the lipid membrane border on the F_O_ rotation.** (A) Schematic picture of the altered lipid membrane regions. The concrete angles are $-69.4^{\circ}$, $-59.1^{\circ}$, $-52.2^{\circ}$, $-48.4^{\circ}$, and $-40.6^{\circ}$ degree from the base to asub137, respectively. (B) The rotational velocity of each setup. The more the lipid membrane region intrudes a-subunit, the slower it rotates. (C) The number of protons moved per one rotation. The number of transported protons decreases as the membrane region expands. Additionally, proton leaks without contributing to the F_O_ rotation when the membrane region locates at the aE162 (asub137).

Supplementary Tables

**Table S1. The number of proton –transfer per rotation.**

| # of proton  per rotation | Transport | | Idle | |
| --- | --- | --- | --- | --- |
|  | IMS$\to$matrix | matrix$\to$IMS | IMS$\to$IMS | matrix$\to$matrix |
| Synthesis | 10.0$\pm$0.1 | 0 | 0 | 0 |
| Hydrolysis | 0 | -10.2$\pm0.15$ | 0.1$\pm$0.038 | 0 |

In the both setups have no leak proton so it was omitted.

**Table S2. The list for Fig. 5.**

|  | pKa | | | pH | | $\Delta\Psi$ (mV) |
| --- | --- | --- | --- | --- | --- | --- |
|  | aE223 | cE59 | aE162 | IMS | matrix |  |
| Base | 6 | 8 | 9 | 7 | 8 | 150 |
| pKa8 | 6 | 8 | 8 | 7 | 8 | 150 |
| pKa7 | 6 | 8 | 7 | 7 | 8 | 150 |
| pH9 | 6 | 8 | 9 | 7 | 9 | 150 |
| pH7 | 6 | 8 | 9 | 7 | 7 | 150 |
| dV300 | 6 | 8 | 9 | 7 | 8 | 300 |
| dV0 | 6 | 8 | 9 | 7 | 8 | 0 |

The red number corresponds to the difference from the base.

Supplementary Movies

**Movies S1 to S2.** The coloring method is same as Fig. 1
